# Quality of care in patients with hypertension: a retrospective cohort study of primary care routine data in Germany

**DOI:** 10.1186/s12875-024-02285-9

**Published:** 2024-02-11

**Authors:** Christoph Strumann, Nicola J. Engler, Wolfgang C. G. von Meissner, Paul-Georg Blickle, Jost Steinhäuser

**Affiliations:** 1 Institute of Family Medicine, University Medical Center Schleswig-Holstein, Campus Lübeck, Ratzeburger Allee 160, 23562 Luebeck, Schleswig-Holstein Germany; 2 Hausärzte Am Spritzenhaus, Family Practice, Baiersbronn, Germany

**Keywords:** Quality indicator, Electronic health record (EHR), Routine data, Primary care

## Abstract

**Background:**

Hypertension is a leading cause of morbidity and mortality if not properly managed. Primary care has a major impact on these outcomes if its strengths, such as continuity of care, are deployed wisely. The analysis aimed to evaluate the quality of care for newly diagnosed hypertension in routine primary care data.

**Methods:**

In the retrospective cohort study, routine data (from 2016 to 2022) from eight primary care practices in Germany were exported in anonymized form directly from the electronic health record (EHR) systems and processed for this analysis. The analysis focused on five established quality indicators for the care of patients who have been recently diagnosed with hypertension.

**Results:**

A total of 30,691 patients were treated in the participating practices, 2,507 of whom have recently been diagnosed with hypertension. Prior to the pandemic outbreak, 19% of hypertensive patients had blood pressure above 140/90 mmHg and 68% received drug therapy (*n* = 1,372). After the pandemic outbreak, the proportion of patients with measured blood pressure increased from 63 to 87%, while the other four indicators remained relatively stable. Up to 80% of the total variation of the quality indicators could be explained by individual practices.

**Conclusion:**

For the majority of patients, diagnostic procedures are not used to the extent recommended by guidelines. The analysis showed that quality indicators for outpatient care could be mapped onto the basis of routine data. The results could easily be reported to the practices in order to optimize the quality of care.

**Supplementary Information:**

The online version contains supplementary material available at 10.1186/s12875-024-02285-9.

## Introduction

Global hypertension prevalence reached 33% among the adult population in 2019, with high blood pressure (BP) being under control in about 23% of treated women and 18% of treated men [1]. Poorly controlled or untreated hypertension has been recognized as a major trigger for cardiovascular events, such as strokes and heart attacks [2, 3]. These diseases are among the world's leading causes of death [4]. In addition, uncontrolled hypertension accounts for about 10% of all health care costs worldwide [5]. Optimizing the medical care of patients with high BP to improve the long-term outcomes and increasing cost-effectiveness represents a key issue in primary health care [6]. Especially since hypertension is the most commonly treated condition in primary care [7] and for the majority of patients with hypertension, primary care physicians were the only healthcare providers [8]. Therefore, many guidelines attempt to provide guidance to primary care physicians [9–12].

An important approach in enhancing diagnosis and treatment to prevent the development of secondary diseases due to hypertension is improving the quality of care. However, to ensure that high-quality care is actually provided, the starting point for measuring quality should be routine data [13].

In Germany, suitable indicators for patient care are provided by the “Quality Indicator System for Ambulatory Care (Qualitätsindikatorensystem für die ambulante Versorgung, QISA)” [14]. The QISA considers diagnostic aspects, such as the frequency of BP measurements, as well as the therapeutic aspects, such as drug therapy, in order to achieve sufficient BP control [15].

However, so far the access to routine care data in Germany is hindered by outdated software interfaces, insufficient software maintenance, organizational and financial burdens imposed by software vendors, and inadequate IT standards [16]. Furthermore, the ambulatory care sector is highly separated from inpatient care and is organized in mostly privately run practices with free provider choice [17]. Therefore, a wide range of electronic health record (EHR) systems are used which are largely inadequate for data extraction [18]. The Supraregional Health Service Research Network (SHRN) was established to enable the analysis of anonymized routine data from GP practices in Germany [19].

The aim of this analysis was to retrospectively evaluate the quality of care of the treatment of patients with newly diagnosed hypertension in routine primary care data by using adapted quality indicators from QISA.

## Methods

### Study design and data collection

In this retrospective cohort study, routine data was extracted from eight private practices in primary care in south-west Germany. The participating practices were recruited from the Supraregional Health Service Research Network (SHRN), Germany. In these practices, 38 general practitioners are currently treating more than 100,000 reasons for encounter in a year. The sample consisted of patients who were treated between 2016 and the outbreak of the pandemic (at the end of the first quarter of 2020, i.e., 2020q1) in one of the participating practices. We applied this inclusion criteria to exclude patients that have visited the practices solely for corona pandemic reasons, e.g., testing and vaccination. In addition to sociodemographic information, data about the practice visits (diagnoses, prescriptions) and laboratory test results, as well as permanent diagnoses the patients have received prior to the sample period, were considered in the analysis. Details about the data processing can be found in Strumann et al. [19].

The analysis concentrated on the quality of care of patients with newly diagnosed with hypertension. For this purpose, patients have been observed for over a year and a half after being diagnosed with hypertension. The considered patients were selected with regard to information stored in the data from the permanent diagnoses for hypertension (ICD 10 diagnoses I10 to I15) [20].

### Quality indicators

We used the 2nd version of QISA indicators from 2020 as the basis for our analysis [15]. The eleven indicators address the process, outcome, and structural quality of primary care of patients diagnosed with hypertension. Due to the available routine data, we focused on five indicators that could be analysed retrospectively. The resulting five indicators will be presented briefly.

#### Process quality

The first quality indicator describes the prevalence and incidence of hypertension in the study population to enable comparability with national and international averages. To identify the respective patients, patients that have received at least one of the ICD 10 diagnoses I10 to I15 (Hypertension) were included. Subdivisions were made according to age, gender, comorbidities, and other diseases to account for sociodemographic characteristics in the evaluation.

Since the outcome and prognosis of hypertension is largely determined by the development of certain comorbidities, such as cardiovascular diseases, the screening of risk factors is particularly important [12]. Therefore, the second considered indicator measures the proportion of newly diagnosed hypertensive patients with a ‘basis diagnostic’. The execution of a basis diagnostic was identified if a documented electrocardiogram (ECG), a blood test, and a physical examination were stored in the EHR during the observation period (i.e., a year and a half after the patient’s first chronical diagnosed hypertension).

In order to successfully treat high BP and thus minimize the risk for secondary diseases, regular monitoring of BP levels is essential [12]. Thus, the third indicator considered all patients with routine BP measurements during the observation period. Both, single BP measurements and 24-h ambulatory blood pressure monitoring (ABPM) were included.

In addition to lifestyle interventions, such as quitting smoking or weight reduction, the guideline-based treatment of hypertension is based on different groups of drugs. The drugs can be prescribed individually or in combination to adequately lower high BP [12]. The fourth indicator describes the number of patients whose hypertension was treated with drugs. The selection of included drugs was performed in accordance with the anatomical therapeutic chemical (ATC) classification [21]. This indicator was divided into one group without any drug therapy, one with only one drug (Monotherapy) and one with a combination of at minimum two drugs (Combination-Therapy).

#### Outcome quality

The risk of cardiovascular events declines with lower blood pressure levels. The incremental benefit of blood pressure lowering, however, decreases as target blood pressure is lowered [22]. At the same time, there is an increased risk of discontinuation due to treatment-related adverse effects in patients seeking blood pressure lowering, which may offset the limited incremental reduction in cardiovascular risk [23]. Therefore, an optimal target BP should balance the cardiovascular risk reduction and the risk of treatment discontinuation. For this reason, many guidelines recommend a general target blood pressure of less than 140/90 mm Hg as the primary outcome [12, 24]. We measured the outcome quality by considering the number of patients for whom the primary treatment outcome was achieved during the observation period.

#### Structural quality

As there was no data available for the evaluation of the structural quality of individual practices within the context of this study, the corresponding indicators from QISA could not be considered. Nevertheless, to address a part of the structural aspect, we performed an additional analysis of individual practice clusters. The aim here was to investigate whether individual patterns regarding diagnostics or the prescription of drugs could be identified among the practices.

### Statistical analysis

Since multiple aspects influence the quality of therapy, we made subdivisions in our analysis with respect to sociodemographic characteristics, the complexity of medical therapy, and the number of used drugs. Due to the influence of the SARS-CoV-2-Pandemic in the beginning of 2020, access to healthcare around the world unexpectedly changed and important care for patients with hypertension was negatively affected [25]. We therefore subdivided patients into groups whose hypertension was diagnosed before and after the outbreak of the pandemic.

The statistical analyses were performed for patients with newly diagnosed hypertension over a period of a year and a half after their first diagnosed hypertension. This period was considered to be the observation period. For the quality indicators measuring process and outcome quality, bivariate analyses were applied by comparing distinct subgroups. In a first step, the prevalence and incidence of hypertension in the study population were computed separately for females and males and for different age groups. The other quality indicators were compared between patients receiving different medication therapies (none, mono and combination) and between patients with a diagnosis before and after the outbreak of SARS-CoV-2. For the former group we categorized patients who received their first diagnosis between 2016 and the first quarter of 2019 (2019q1) so that the observation period (a year and a half from the diagnosis) was before the outbreak of the pandemic in 2020q1 and as such avoided any overlap with the outbreak of the pandemic. For the latter group, patients were included if they received their first diagnosis between 2020q2 and 2021q1, leading to an observation period until the end of the sample (2022q1).

Differences of the considered variables were tested by means of t-test and Analysis of Variance (ANOVA) or ${\chi }^{2}$-test (if the respective variable was nominally scaled). The importance of individual practice clusters among the considered quality indicators were analyzed by estimating separate multivariate regression models for each indicator (logistic models for binary variables and linear models for numerical variables). Using likelihood ratio (LR) tests, the improvements in the model fit after the inclusion of random effects at the physician level and the Intraclass Correlation Coefficients (ICCs) were used to assess the importance of individual practice clusters [26]. The patients’ sex, age, multimorbidity and specific comorbidities, as well as secondary diseases were included as control variables. This analysis was concentrated on patients that have received their diagnosis before the pandemic outbreak. Multiple imputation was used to check the robustness of the data (see Appendix 1, Supplemental Material). The imputation models included complete variables that served as explanatory variables.

## Results

### Sample characteristics

In total, between 2016 and the first quarter of 2022, 30,691 patients visited one of the participating practices. Females were slightly more represented (55.2%). While 2023 patients had been diagnosed with high BP between 2016-2019q, 484 (1.6%) patients received their hypertension diagnosis after the first quarter of 2020. In Table 1, the sample characteristics were displayed for all patients that have visited one of the practices during the sample period before the pandemic, and for patients that have been diagnosed with hypertension during the sample period (2016-2022q1) before (2016-2019q1) and after (2020q2 and 2021q1) the outbreak of the pandemic.

**Table 1:** Patient characteristics

Variable		Hypertensive patients with diagnosis between		
	all	2016-2019q1	2020q2-2021q1	
	*n* = 30,691 (100%)	*n* = 2023 (6.6%)	*n* = 484 (1.6%)	*p*-value^f^
*Sociodemographic (at the beginning of the disease)*				
Female, n(%)	16,929(55.2)	1052(52.0)	259(53.5)	0.0056
Male, n(%)	13,751(44.8)	971(48.0)	225(46.5)	0.0049
Age^a^, µ	53.7	67.4	62.5	< 0.0001
Age at the time of diagnosis, µ	-	63.1	60.9	< 0.0001
BMI (kg/m^2^), µ/N	27.3/1643^b^	29.0/1511^b^	29.3/378^b^	< 0.0001
*Health Status (during the sample period)*				
Number of diagnoses (ICD-Chapter) per practice visit, µ	2.0	2.1	2.8	< 0.0001
Number of diagnoses for chronical diseases^c^ per practice visit, µ	0.8	1.3	2.5	< 0.0001
Multimorbid^d^, n(%)	5547(18.1)	685(33.9)	192(39.7)	< 0.0001
*Specific comorbidities/*other diseases* (during the sample period)*				
Obesity (E66.0, E66.9), n(%)	2091(6.8)	281(13.9)	47(9.7)	< 0.0001
Diabetes mellitus 1 and 2 (E10, E11, E14, R73.0), n(%)	4065(13.2)	530(26.2)	119(24.6)	< 0.0001
Cardiovascular disease (I20, I21, I24, I25, I70, I63-I66), n(%)	3554(11.6)	493(24.4)	107(22.1)	< 0.0001
Mental and behavioral disorders (F00-F99), n(%)	7275(23.7)	658(32.5)	218(45.0)	< 0.0001
others^e^, n(%)	3002(9.8)	255(12.6)	63(13.0)	< 0.0001

µ: mean; N: number of observations; ^a^The most recent entry was used. ^b^Missing values due to non-performed examination. ^c^Chronical Diagnoses were only counted if they were coded in at least 3 quarters within the sample period [20]. ^d^Multimorbid patients were identified, if more than two chronical diagnoses [27] were coded in at least 3 quarters within the sample period [20]. ^e^elevated blood-pressure without hypertensive diagnosis (R03.0), hyrotoxicosis (E05.0), hypothyroidism (E03.0), subcl. iodine deficiency hypothyroidism (E02), Autoimmune thyroiditis (E06.3). ^f^Bonferroni corrected p-value for testing for differences between groups of patients

18.1% of all 30,691 patients were diagnosed with multimorbidity, which is defined as having two or more chronic diseases [27]. This percentage was almost twice as high for patients with hypertension. Patients with hypertension were also observed to have higher percentages for several comorbidities (e.g., cardiovascular disease, mental and behavioural disorders) and secondary diseases (e.g., obesity, diabetes mellitus). Patients who were diagnosed after the start of the pandemic had a higher burden of disease as measured by the number of diagnoses, chronic diseases/conditions, multimorbidity and specific comorbidities/secondary diseases.

### Incidence and prevalence

Table 2 shows the annual incidence and prevalence rates for distinct subgroups of patients before and after the outbreak of the pandemic. Prior to the pandemic outbreak, on average, 27.9 patients (per thousand patients) have been newly diagnosed as hypertensive per year with an average prevalence rate of 27.3%. The prevalence and incidence rates were slightly larger for males. Both the prevalence and incidence rates increased with the age of patients. For all subgroups, the incidence rates were lower, while the prevalence rates were higher after the outbreak of the pandemic.

**Table 2:** Annual incidence and prevalence

Subgroup		Prior pandemic outbreak	After pandemic outbreak	*p*-value* (prior vs. after)
	Incidence/Prevalence	2016-2020q1	2020q2-2022q1	
all	Incidence^a^	27.9	19.1	< 0.0001
n(%) = 30,691(100)	Prevalence^b^	27.3	29.3	< 0.0001
*sex differences*				
Female	Incidence^a^	26.4	18.4	< 0.0001
n(%) = 16,929(55.2)	Prevalence^b^	26.7	28.5	< 0.0001
Male	Incidence^a^	29.8	20.0	< 0.0001
n(%) = 13,751(44.8)	Prevalence^b^	28.2	30.2	< 0.0001
*p*-value*: Sex differences	Incidence	< 0.0001	< 0.0001	
	Prevalence	< 0.0001	< 0.0001	
Age^c^ differences				
< 35	Incidence^b^	4.4	3.8	< 0.0001
n(%) = 9172(29.9)	Prevalence^b^	2.5	3.2	0.0240
35–64	Incidence^a^	29.5	24.4	< 0.0001
n(%) = 14,159(46.1)	Prevalence^b^	25.0	28.5	< 0.0001
65–79	Incidence^a^	72.4	48.9	< 0.0001
n(%) = 5258(17.1)	Prevalence^b^	59.7	63.4	< 0.0001
> 80	Incidence^a^	85.6	52.7	< 0.0001
n(%) = 2102(6.8)	Prevalence^b^	69.8	72.2	< 0.0001
*p*-value*: Age differences	Incidence	< 0.0001	< 0.0001	
	Prevalence	< 0.0001	< 0.0001	

^*^Bonferroni corrected. ^a^Incidence: number of newly diagnosed hypertensive patients per thousand patients that visited a practice during the sample period divided by the number of considered years (i.e., 4.25 for the prior pandemic outbreak period and 2 for the post outbreak period); ^b^Prevalence: number of hypertensive patients in % of all patients that visited a practice during the sample period divided by the number of considered years. ^c^The age of the patients at the beginning of the sample period (i.e., 2016) was considered

### Diagnostic and therapeutic indicators

Table 3 shows pre-pandemic diagnosis, therapy and BP data for patients treated with different types of medical therapies, i.e., no medication, mono- or combination therapy.

**Table 3:** Newly diagnosed hypertensive patients (2016-2019q1) with different types of medical therapies

Variable	None	Mono	Combination	*p*-value^a^
	n(%) 651(32.2)	n(%) 458(22.6)	n(%) 914(45.2)	
*BP measurement during first 1.5 years of disease*				
BP measured, n (%)	128(19.7)	364(79.5)	782(85.6)	< 0.0001^b^
number of BP measurements, µ	0.4	2.3	3.6	< 0.0001
BP measured at least 3 times, n (%)	35(5.4)	162(35.4)	501(54.8)	< 0.0001^b^
ABPM, n (%)	18(2.8)	77(16.8)	160(17.5)	< 0.0001^b^
Basis diagnostic, n (%)	12(1.8)	35(7.6)	96(10.5)	< 0.0001^b^
Health Check-up, n (%)	52(8.6)	146(41.5)	336(51.3)	< 0.0001^b^
*Therapy during sample period*				
Number of different drugs, µ	-	2.7	8.2	< 0.0001
Number of different ATCs, µ	-	1.0	2.7	< 0.0001
Number of different APIs, µ	-	1.1	3.2	< 0.0001
*Most prevalent APIs*				
Ramipril, (%)	-	206(45.0)	429(46.9)	< 0.0001^b^
Bisoprolol, (%)	-	58(12.7)	294(32.2)	< 0.0001^b^
Amlodipine, (%)	-	25(5.5)	263(28.8)	< 0.0001^b^
Candesartan, (%)	-	51(11.1)	201(22.0)	< 0.0001^b^
Torasemide^c^, (%)	-	32(7.0)	165(18.1)	< 0.0001^b^
Metoprolol, (%)	-	26(5.7)	151(16.5)	< 0.0001^b^
*BP measures at the beginning of disease*				
Systolic mmHg, µ/N	137.8/92	142.7/275	139.2/564	> *0.999*
Diastolic mmHg, µ/N	81.5/92	84.5/275	80.8/564	0.0016
> 140/90 mmHg, n/N(%)	23/92(25.0)	95/275(34.5)	121/564(21.5)	0.0059^b^
< 140/90 mmHg, n/N(%)	38/92(41.3)	99/275(36)	250/564(44.3)	> *0.999*^b^
*BP measures after one year of diagnosis*				
Systolic, µ/N	134.2/45	136.6/160	137.5/517	> *0.999*
Diastolic, µ/N	80.6/45	81.3/160	80.1/517	> *0.999*
> 140/90 mmHg, n/N(%)	10/45(22.2)	33/160(20.6)	95/516(18.4)	> *0.999*^b^
< 140/90 mmHg, n/N(%)	23/45(51.1)	75/160(46.9)	249/516(48.3)	> *0.999*^b^
*BP measures differences*				
Systolic (Δ), µ/N	1.2/20	3.2/95	-0.2/350	> *0.999*
Diastolic (Δ), µ/N	-1.9/20	0.2/95	0.1/350	> *0.999*

*BP* Blood pressure, *ABPM* 24-h Ambulatory Blood Pressure Monitoring, *API* Active pharmaceutical ingredient, *ATC* Anatomical therapeutic chemical classification code,  Monotherapy: prescription of drugs with one API; Combination therapy: prescription of drugs with more than one API; µ: mean; N: number of observations with non-missing data; ^a^Bonferroni corrected. Italicized records indicate *p*-values > 0.05. ^b^χ^2^ test. ^c^ Torasemide is generally not used primarily to lower blood pressure, but to treat fluid retention due to heart, kidney, or liver disease

While one-third of the newly diagnosed patients (*n* = 651) did not receive any hypertensive drugs, almost every second patient (*n* = 914) was treated with drugs with more than one active pharmaceutical ingredient (API) (combination-therapy). The differences in the application of blood measurements between mono- and combination-therapy patients were rather small during the first 1.5 years of their disease (mono: 79.5%, combination: 85.6%). In contrast, patients who have not received any medication therapy have significantly smaller frequencies; BP measurement was performed in 19.7% of patients. Naturally, the number of prescribed drugs, API and ATC classification codes were larger in the group of patients with combination-therapy. The most prevalent APIs were Ramipril, Bisoprolol, Amlodipine, Candesartan and Torasemide. At the beginning of the hypertension disease, the average systolic as well as the diastolic BP were significantly higher for the patients treated with monotherapy (142.7/84.5 mmHg) and smaller for patients that did not receive any medication (137.8/81.5 mmHg). (The latter statistic was, however, based on only 92 observations.) More than 30% of the patients with monotherapy had a BP over 140/90 mmHg. For patients with combination therapy, this percentage was 21.5% and for patients without medication therapy 25%. After one year, differences in the BP measures between the groups of patients were insignificant.

### Effect of the pandemic on the quality of care

In Table 4, the same data are shown for subgroups of patients that were diagnosed before the outbreak of the pandemic and patients who received their diagnosis after the outbreak of the pandemic.

**Table 4:** Newly diagnosed hypertensive patients before and after the pandemic outbreak

Variable	2016-2019q1	2020q2-2022q1	*p*-value^a^
	*n* = 2023	*n* = 484	
*BP measurement during first 1.5 years of disease*			
BP measured, n(%)	1274(63.0)	421(87.0)	< 0.0001^b^
number of BP measurements, µ	2.3	2.7	*0.089*
BP measured at least 3 times, n(%)	698(34.5)	203(41.9)	0.048^b^
ABPM, n(%)	255(12.6)	90(18.6)	0.013^b^
Basis diagnostic, n(%)	143(7.1)	53(11.0)	*0.094*^b^
Health Check-up, n(%)	534(35.3)	163(38.6)	*0.100*^b^
*Therapy during sample period*			
No medical therapy, n(%)	651(32.2)	75(15.5)	< 0.0001^b^
Mono-therapy, n(%)	458(22.6)	131(27.1)	*0.86*^b^
Combination-therapy, n(%)	914(45.2)	277(57.2)	< 0.0001^b^
Number of different drugs, µ	4.3	5.4	0.0012
Number of different APIs, µ	1.7	2.2	< 0.0001
Number of different ATCs, µ	1.4	1.8	< 0.0001
*BP measures at the beginning of disease*			
Systolic, µ/N	140.1/931	145.4/319	0.0024
Diastolic, µ/N	82.0/932	86.0/319	< 0.0001
> 140/90 mmHg, n/N(%)	239/931(25.7)	123/319(38.6)	0.0003^b^
< 140/90 mmHg, n/N(%)	387/931(41.6)	102/319(32.0)	*0.054*^b^
*BP measures after one year of diagnosis*			
Systolic, µ/N	137.1/722	140.5/165	*0.86*
Diastolic, µ/N	80.4/722	82.8/165	*0.11*
> 140/90 mmHg, n/N(%)	138/721(19.1)	35/165(21.2)	> *0.999*^b^
< 140/90 mmHg, n/N(%)	347/721(48.1)	75/165(45.5)	> *0.999*^b^
*BP measures differences*			
Systolic (Δ), µ/N	0.6/465	2.2/100	> *0.999*
Diastolic (Δ), µ/N	0.0/465	0.7/100	> *0.999*

*BP* Blood pressure, *ABPM* 24-h Ambulatory Blood Pressure Monitoring, *API* Active pharmaceutical ingredient, *ATC* Anatomical therapeutic chemical classification code,  *Monotherapy *Prescription of drugs with one API, *Combination therapy *Prescription of drugs with more than one API, *µ *mean, *N *Number of observations with non-missing data; ^a^Bonferroni corrected. Italicized records indicate *p*-values > 0.05. ^b^χ^2^ test

After the outbreak of the pandemic, the BP was measured mostly in the first 1.5 years of the disease (63.0% vs. 87.0%). ABPMs also increased, however, the difference was smaller. A basic diagnostic (consisting of laboratory test, ECG and physical examination) was performed in 7.1% to 11% of patients, and a complete health check-up was performed in 35.3% to 38.6% of patients. While the percentage of patients receiving monotherapy was rather stable, the percentage of patients receiving no medical therapy has dropped from 32.2% to 15.5%. Instead, the frequency of combination-therapy has increased after the outbreak. Increases were also observed for the number of different prescribed drugs, as well as for the number of APIs and ATC classification codes. After the outbreak of the pandemic, the average systolic and the diastolic BP were significantly higher (140.1/82.0 mmHg vs. 145.4/86.0 mmHg), as well as the percentage of patients with a BP over 140/90 mmHg (25.7% vs. 38.6%).

### Practice clusters

Table 5 provides the results of the analysis of the importance of individual practice clusters among the considered quality indicators. The relative change in the model fit (log likelihood value) is shown for each variable if random intercepts on the practice level (multi-level) are additionally included in the regression models. Furthermore, the table provides the estimated ICCs with the Bonferroni corrected 95% confidence interval.

**Table 5:** Practice clusters among newly diagnosed hypertensive patients prior to the pandemic outbreak

Variable		ΔFit^a^ (in %)	P-value (LR-Test^c^)	Intraclass Correlation (in %)		
	Model			estimate	95%-CI^b^	
					lower	upper
*BP measurement during first 1.5 years of disease*						
BP measured	logit	28.2	< 0.0001	54.4	29.0	77.8
Number of BP measurements	linear	3.3	< 0.0001	18.0	7.0	39.1
BP measured at least 3 times	logit	13.8	< 0.0001	54.4	24.4	81.5
ABPM	logit	23.6	< 0.0001	79.7	40.3	95.8
Basis diagnostic	logit	24.4	< 0.0001	75.2	35.8	94.3
Health Check-up	logit	28.9	< 0.0001	61.1	33.2	83.2
*Therapy during sample period*						
No medical therapy	logit	21.8	< 0.0001	40.2	18.8	66.1
Mono-therapy	logit	3.8	< 0.0001	16.9	6.0	39.5
Combination-therapy	logit	10.4	< 0.0001	28.9	12.0	54.8
Number of different drugs	linear	2.8	< 0.0001	18.6	7.3	40.1
Number of different APIs	linear	4.1	< 0.0001	19.3	7.6	41.1
Number of different ATCs	linear	4.9	< 0.0001	20.6	8.2	43.1
Number of different ACE inhibitors	linear	1.5	< 0.0001	8.8	3.1	22.6
*Outcome quality*						
< 140/90 mmHg (during the first year)	logit	0.0	> *0.999*	0.1	0.0	100
< 140/90 mmHg (after one year)	logit	0.0	*0.92*	1.7	0.0	11.2

*n* = 2023; 8 practices; 252.9 hypertensive patients were treated on average by a practice, *BP* Blood pressure, *ABPM* 24-h Ambulatory Blood Pressure Monitoring, *API* Active pharmaceutical ingredient, *ATC* Anatomical therapeutic chemical classification code, *Monotherapy *Prescription of drugs with one API, *Combination therapy *Prescription of drugs with more than one API, *logit *Logistic regression model, *linear *Linear regression model; ^a^ΔFit is the relative change in model fit between a model with covariables only and a multilevel model measured by the log-likelihood values of the respective estimated models; ^b^Bonferroni corrected 95% confidence interval. ^c^Bonferroni corrected *p*-value of a Likelihood Ratio test. Italicized records indicate *p*-values > 0.05

In total, the eight practices treated on average 289 of the 2023 newly diagnosed hypertensive patients (prior the pandemic outbreak). Practice clusters seem to play a more important role in the diagnostic than for medical therapy. By accounting for physician random effects, the fit of a logistic regression model of conducting a blood measurement during the first 1.5 years of the disease, increased by more than 28%. In this model, the estimated intra-class coefficients indicate that, conditional on the covariates, more than 54.4% of total variation in conducting a blood measurement could be explained by the individual practices. For the other diagnostic measures, this proportion exceeds 54%. Regarding ABPM, this proportion is almost 80%. The explanatory power of the practice level of the medical therapy is much lower (8.8% to 40.2%). For the outcome quality, the respective estimated intra-class coefficients are near to zero and insignificant.

## Discussion

The present study examines the quality of primary care for patients with hypertension using routine data from eight primary care practices in Germany.

Our data reveal a prevalence of 27.3% prior to the pandemic. This number, as well as the finding that men have a higher prevalence, are in line with findings from other German studies, e.g. [28]. Similar to other studies, this study also shows that the probability of developing hypertension increases with age [29]. When comparing these groups before and during the pandemic, there is a slight but significant decrease in age at diagnosis during the pandemic and in addition, there is a significantly lower average age in this group. This can be explained by a higher health awareness among younger patients during the pandemic, which, for example, could have led to more incidental findings of high BP. This assumption can be supported by the sharp increase in BP measurements performed after the outbreak (from 63 to 87%), which can be explained by the fact that hypertension was considered a risk factor for a severe course of Covid-19 [30].

The data of our sample suggest that the number of diagnoses for chronical diseases has almost doubled for patients with hypertension after the outbreak of the pandemic. Nearly 40% of the patients with hypertension have been identified as multimorbid after the outbreak. Every fourth patient with hypertension suffered additionally from diabetes and cardiovascular diseases. A possible explanation for the increase in chronic diseases is that the patients might have changed their diet or physical activity because of restrictions imposed by pandemic containment measures or the anxiety and stress they caused [31]. In our data, we also observed a slight increase in mean BMI from 29.0 to 29.3 after the pandemic. However, this rise as well as the increase in chronic diseases may also be due to a selection effect because a higher health awareness during the pandemic. However, multimorbid patients are at a particular risk of cardiovascular events [32]. The sharp increase of conducted BP measurements after the outbreak might be a helpful tool for proactively avoiding such deteriorations. Improvements in the risk profile of patients can also be achieved by motivating them to adopt healthier lifestyles [33]. This is in line with the recommendations of the guideline for cardiovascular prevention of the German College of General Practice and Family Physicians (DEGAM) which says that all patients with arterial hypertension, lifestyle modification interventions should form the basis of antihypertensive therapy [12]. Furthermore, there was a large increase in mental illnesses during the pandemic. This is consistent with other studies that have observed a general increase in mental health disorders, such as depression, and suggests a negative impact of the COVID-19-Pandemic on people’s mental health [34]. Similar to other studies, our data suggest an increase in the BP measured at the beginning of the disease after the pandemic outbreak [35].

In the first 1.5 years of the disease, prior to the pandemic, the BP was measured in 63% of cases. The DEGAM guideline for cardiovascular prevention recommends to confirm the diagnosis of hypertension by measuring the BP. Additionally, three measurements should be taken on at least two different days [12]. Our data suggest that only one in three patients had their BP measured at least three times during the first 1.5 years of the disease. Other studies find even lower numbers for Germany, e.g., below 25% [36]. In general, the prognostic power of such office-based BP measurements for the risk of cardiovascular disease events is considered to be lower in comparison with home-based measurement or ABPM, e.g. [37]. The frequency of using ABPM is even lower (12.6%-18.6%). However, recent technological developments also allow a direct electronic transmission of self-measured blood pressures to the EHR [38]. Future studies should incorporate these additional measures and distinguish between office and home based measurements.

Prior to the pandemic, less than half of the patients received combination therapy (45.2%), while 22.6% received monotherapy and around a third did not receive any medication. Other studies have documented similar rates. Based on two million patients from statutory health insurance data from 2011 to 2013 in Germany, combination therapy was prescribed for 40.6% of patients with a new diagnosis of hypertension after one year, while therapy was not prescribed in 21.7% of cases [39]. A nation-wide survey obtained a similar number for combination therapy (38.2%) [36]. The German DEGAM guideline for cardiovascular prevention suggests to start therapy with monotherapy and combination therapy [12]. The latter is recommended when treatment with monotherapy at an appropriate dose still results in BP 20/10 mmHg above the target [40]. According to QISA, monotherapy may be considered in individuals with systolic BP below 150 mmHg and low cardiovascular risk, as well as in patients over the age of 80 years or individuals who are frail [15]. In general, antihypertensive therapy should be continued indefinitely because BP reduction to < 140/90 mmHg can usually not be maintained after discontinuation [41]. However, medical therapy could have also undesirable effects in older adults with several chronic diseases [42] and should be considered depending on comorbidities and other medications [12]. For younger and more healthy patients with a general lower cardiovascular risk, medical therapy might not always be indicated as the number needed to treat is disproportionately high for blood pressure lowering and shared decision making could result in a conscious decision not to lower blood pressure treatment [43]. Although the reported rates of medication therapy are consistent with national findings from related studies, further information on other medications and comorbidities should be considered to assess the quality of care.

Our findings suggest that prior to the pandemic, about one fourth of the patients with a newly diagnosed hypertension have a BP that, at the beginning of the disease, was over 140/90 mmHg. Slightly more than 40% of the patients had a BP in the controlled range (i.e., below 140/90 mmHg). These numbers are also very similar to the findings of other studies, where e.g. 40.8% of treated patients had a BP in the controlled range [36]. After one year of diagnosis, almost half of the patients achieved normotension. In related studies, this percentage is a bit higher (i.e., 57.3%) [44].

Furthermore, our results suggest that the individual practice style has a significant influence on the considered indicators for process quality. The very high estimate of the intraclass correlation coefficient for ABPM, suggesting that 80% of the total variation in conducting an ABPM could be explained by individual practices, may be explained by the fact that not each practice has access to an ABPM device. However, also the estimates for the other process quality indicators, such as whether a BP is measured or the conduction of a basis diagnostic are rather high, up to 75.2%. The importance of practice level clusters for the medical therapy is only half of the size, but also substantial. Interestingly, for the outcome quality, physician clusters do not seem to play a role. However, these findings underscore that there is great potential to improve process quality of care by changing individual physician behaviors. This could be achieved by different forms of interventions, e.g. educational [45] or so-called best practice alerts, i.e., clinician decision support tools available in the EHR to remind the physician to measure BP [46] or doing prescriptions [47] during practice visits.

### Strength and limitations

Most related studies use survey data [36, 48] or routine data that is extracted from a specific health care institution or from a specific health insurance [39]. All these sources have different drawbacks. Survey-based studies are prone to selection bias [49]. In contrast, routine data provide reliable information that avoids selection or recall bias [50]. Studies analysing routine data from only one clinic or a specific health insurance have limited representativeness [51, 52]. A strength of this study is the extraction and analysis of routine data from eight different primary care practices. Although the practices are all located in south-west Germany, they cover a broader population than a specific hospital, as hypertension is mostly treated in primary care [7]. Looking at the sociodemographic data of our study population, women are slightly overrepresented. This, however, corresponds to the statistical distribution of the total population of Germany [53]. In addition, the data used in this study allow for matching of diagnostic tests, treatment decisions (here prescriptions), and patient outcomes. In contrast, health insurances can only provide data used for billing purposes.

Nevertheless, the limitation of non-uniform documentation patterns in the different practices, which may have led to loss or non-acquisition of data, must be taken into account. For example, limitations in the documentation of home and self-measurements make it impossible to distinguish between office and self-measurements. However, this could play a crucial role when evaluating the DEGAM guideline recommendation to take three measurements on at least two different days. Further, in some of the practices, BP might be measured before a blood sample is taken. However, for this purpose, many patients come to the practice fasting even without taking their medications in the morning, and therefore might have a higher BP than they would have usually. Changes in the incentives regarding complete health check-ups or the number of ABPM devices per practice might have some influence on routine care data.

Our data suggest a prevalence of 27.3% if patients with hypertension were identified by relying on diagnoses for hypertension (ICD 10 diagnoses I10 to I15). Data from related studies estimates a similar prevalence rate [54]. However, ICD-10 codes are known to be subject to misclassification [55]. Furthermore, if antihypertensive drug prescriptions and abnormal blood pressure were additionally used to identify patients with hypertension, the prevalence increases [56].

## Conclusion

The analysis has shown that quality indicators can be mapped for outpatient care on the basis of routine data, without extra effort for ongoing practice operations. The results of the individual quality indicators could be provided, e.g., in the form of a dashboard in the electronic health record system for practitioners to optimize the quality of care. In addition, the research infrastructure enables the analysis of effects of external events (such as health policy measures or a pandemic) on the quality of outpatient care.

### Supplementary Information

**Additional file 1. Appendix 1.** Multiple imputation procedure. **Table S1**. Number of Missing Values and Multiple Imputation. **Appendix 2. **STROBE Statement—checklist of items that should be included in reports of observational studies. 

## Data Availability

The data sets generated and analysed in the current study are not publicly available due to ethical or privacy reasons. However, data are available from the corresponding author on reasonable request with the permission from the individual practices of the SHRN.
