# Supplementary material for: Quality of care in patients with hypertension: a retrospective cohort study of primary care routine data in Germany
Source: BMC Prim Care. 2024 Feb 11;25:54. doi: 10.1186/s12875-024-02285-9 (PMC10859029; doi:10.1186/s12875-024-02285-9)
Supplement: Supplementary file 1 — Additional file 1. Appendix 1. Multiple imputation procedure. Table S1. Number of Missing Values and Multiple Imputation. Appendix 2. STROBE Statement—checklist of items that should be included in reports of observational studies. [file 12875_2024_2285_MOESM1_ESM.docx]

**SUPPLEMENTAL MATERIAL**

**Quality of Care in Patients with Hypertension: a retrospective cohort study of primary care routine data in Germany**

Authors: Christoph STRUMANN, Nicola J. ENGLER, Wolfgang C. G. VON MEIßNER, Paul-Georg BLICKLE, Jost STEINHÄUSER

**Corresponding Author**

Christoph Strumann

Institute of Family Medicine, University Medical Center Schleswig-Holstein, Campus Lübeck

Ratzeburger Allee 160, 23562, Lübeck, Germany

Tel +49 451 3101 8005

Fax +49 451 3101 8004

E-Mail [c.strumann@uni-luebeck.de](mailto:c.strumann@uni-luebeck.de)

**Appendix 1. Multiple imputation procedure**

Data that have been measured in the practice were missing. This includes BMI in Table 1 and BP measurements at the beginning of disease and one year after in Table 3 and Table 4 (i.e., systolic, diastolic, >140/90 mmHg, <140/90 mmHg), as well as the differences between these time points (Systolic (Δ) and Diastolic (Δ)).

We assumed that data were missing at random enabling us to use other variables in the dataset to predict the missing values by chained equations (MICE). Numeric variables were imputed using linear regression models and binary variables were imputed using logistic regression models. For both variables, we used 20 imputed datasets. The results of the imputed datasets were consolidated into one result by averaging the individual estimates for each of the imputed datasets. To calculate p-values, standard errors were extracted from the covariance matrix, i.e., a combination of the within-imputation variance and the between-imputation variance [1].

Table S1 provides the absolute and relative number of missing observations for the BP measurement variables as well as the p-values that are based on multiple imputation. In general, the multiple imputation procedure confirms the significance level for the differences between the considered subgroups for all variables. (This also applies for BMI, not shown in Table S2.) Exceptions are the number of patients with a BP >140/90 mmHg in Table 3 and for the average systolic BP in Table 4. Both were measured at the beginning of disease.

**Reference**

1. White IR, Royston P, Wood AM. Multiple imputation using chained equations: Issues and guidance for practice. Stat Med. 2011;30(4):377-399. doi: https://doi.org/10.1002/sim.4067

**Table S1: Number of Missing Values and Multiple Imputation**

| Variable | missing values | p-value^a^ | |
| --- | --- | --- | --- |
|  | n/(%) | Table 3 | Table 4 |
| *BP measures at the beginning* *of disease* |  |  |  |
| Systolic | 1257(50.1) | 0.64 | 0.15^b^ |
| Diastolic | 1256(50.1) | 0.019 | 0.0007 |
| >140/90 mmHg | 1257(50.1) | 0.087^b^ | 0.021 |
| <140/90 mmHg | 1257(50.1) | >0.999 | >0.999 |
| *BP measures after one year of diagnosis* |  |  |  |
| Systolic | 1620(64.6) | >0.999 | >0.999 |
| Diastolic | 1620(64.6) | >0.999 | >0.999 |
| >140/90 mmHg | 1621(64.7) | >0.999 | >0.999 |
| <140/90 mmHg | 1621(64.7) | >0.999 | >0.999 |
| *BP measures differences* |  |  |  |
| Systolic (Δ) | 1942(77.5) | >0.999 | >0.999 |
| Diastolic (Δ) | 1942(77.5) | >0.999 | >0.999 |

BP: blood pressure; n: number of observations with missing data; ^a^Based on Multiple Imputation and Bonferroni corrected. ^b^Difference to the significance level (p<0.05) that is based on observations with non-missing data.

**Appendix 2.** STROBE Statement—checklist of items that should be included in reports of observational studies

|  | Item No. | Recommendation | Page  No. | Relevant text from manuscript |
| --- | --- | --- | --- | --- |
| **Title and abstract** | 1 | (*a*) Indicate the study’s design with a commonly used term in the title or the abstract | p.1, p.2 | Lines 1, 31 |
|  |  | (*b*) Provide in the abstract an informative and balanced summary of what was done and what was found | p.2 | Lines 31-46 |
| Introduction | | | |  |
| Background/rationale | 2 | Explain the scientific background and rationale for the investigation being reported | p.3-4 | Lines 69-93 |
| Objectives | 3 | State specific objectives, including any prespecified hypotheses | p.4 | Lines 94-96 |
| Methods | | | |  |
| Study design | 4 | Present key elements of study design early in the paper | p.4-5 | Lines 99-116 |
| Setting | 5 | Describe the setting, locations, and relevant dates, including periods of recruitment, exposure, follow-up, and data collection | p.4-5 | Lines 99-116 |
| Participants | 6 | (*a*) *Cohort study*—Give the eligibility criteria, and the sources and methods of selection of participants. Describe methods of follow-up  *Case-control study*—Give the eligibility criteria, and the sources and methods of case ascertainment and control selection. Give the rationale for the choice of cases and controls  *Cross-sectional study*—Give the eligibility criteria, and the sources and methods of selection of participants | p.4-5 | Lines 99-116 |
|  |  | (*b*) *Cohort study*—For matched studies, give matching criteria and number of exposed and unexposed  *Case-control study*—For matched studies, give matching criteria and the number of controls per case | NA | NA |
| Variables | 7 | Clearly define all outcomes, exposures, predictors, potential confounders, and effect modifiers. Give diagnostic criteria, if applicable | p.5-8 | Lines 119-192 |
| Data sources/ measurement | 8* | For each variable of interest, give sources of data and details of methods of assessment (measurement). Describe comparability of assessment methods if there is more than one group | p.5-8 | Lines 119-192 |
| Bias | 9 | Describe any efforts to address potential sources of bias | p.8, p.2-3 (Appendix 1) | Lines 192-195, 16-34  (Appendix 1) |
| Study size | 10 | Explain how the study size was arrived at | p.4-5 | Lines 99-111 |

Continued on next page

| Quantitative variables | 11 | Explain how quantitative variables were handled in the analyses. If applicable, describe which groupings were chosen and why | p.7-8 | Lines 163-195 |
| --- | --- | --- | --- | --- |
| Statistical methods | 12 | (*a*) Describe all statistical methods, including those used to control for confounding | p.7-8 | Lines 163-195 |
|  |  | (*b*) Describe any methods used to examine subgroups and interactions | p.7-8 | Lines 163-195 |
|  |  | (*c*) Explain how missing data were addressed | p.8, p.2-3 (Appendix 1) | Lines 192-195, 16-34  (Appendix 1) |
|  |  | (*d*) *Cohort study*—If applicable, explain how loss to follow-up was addressed  *Case-control study*—If applicable, explain how matching of cases and controls was addressed  *Cross-sectional study*—If applicable, describe analytical methods taking account of sampling strategy | NA | NA |
|  |  | (*e*) Describe any sensitivity analyses | p.8, p.2-3 (Appendix 1) | Lines 192-195, 16-34  (Appendix 1) |
| Results | | | | |
| Participants | 13* | (a) Report numbers of individuals at each stage of study—eg numbers potentially eligible, examined for eligibility, confirmed eligible, included in the study, completing follow-up, and analysed |  | Table 1 |
|  |  | (b) Give reasons for non-participation at each stage |  | NA |
|  |  | (c) Consider use of a flow diagram |  | NA |
| Descriptive data | 14* | (a) Give characteristics of study participants (eg demographic, clinical, social) and information on exposures and potential confounders |  | Table 1 |
|  |  | (b) Indicate number of participants with missing data for each variable of interest | p.2-3 (Appendix 1) | Lines 16-34  Appendix 1 |
|  |  | (c) *Cohort study*—Summarise follow-up time (eg, average and total amount) | NA | NA |
| Outcome data | 15* | *Cohort study*—Report numbers of outcome events or summary measures over time |  | Tables 1-4 |
|  |  | *Case-control study—*Report numbers in each exposure category, or summary measures of exposure |  |  |
|  |  | *Cross-sectional study—*Report numbers of outcome events or summary measures |  |  |
| Main results | 16 | (*a*) Give unadjusted estimates and, if applicable, confounder-adjusted estimates and their precision (eg, 95% confidence interval). Make clear which confounders were adjusted for and why they were included |  | Table 5 |
|  |  | (*b*) Report category boundaries when continuous variables were categorized |  | NA |
|  |  | (*c*) If relevant, consider translating estimates of relative risk into absolute risk for a meaningful time period |  | NA |

Continued on next page

| Other analyses | 17 | Report other analyses done—eg analyses of subgroups and interactions, and sensitivity analyses | p.2-3 (Appendix 1) | Lines 16-34  Appendix 1 |
| --- | --- | --- | --- | --- |
| Discussion | | | | |
| Key results | 18 | Summarise key results with reference to study objectives | p.11 | Lines 280-281 |
| Limitations | 19 | Discuss limitations of the study, taking into account sources of potential bias or imprecision. Discuss both direction and magnitude of any potential bias | p.13 | Lines 349-369 |
| Interpretation | 20 | Give a cautious overall interpretation of results considering objectives, limitations, multiplicity of analyses, results from similar studies, and other relevant evidence | p.14 | Lines 372-376 |
| Generalisability | 21 | Discuss the generalisability (external validity) of the study results | p.13 | Lines 354-369 |
| Other information | |  | | |
| Funding | 22 | Give the source of funding and the role of the funders for the present study and, if applicable, for the original study on which the present article is based | p.15 | Lines 382-384 |

*Give information separately for cases and controls in case-control studies and, if applicable, for exposed and unexposed groups in cohort and cross-sectional studies.
